# Effects of Temperature Stress and Aquarium Conditions on the Red Macroalga *Delisea pulchra* and its Associated Microbial Community

**DOI:** 10.3389/fmicb.2016.00161

**Published:** 2016-02-18

**Authors:** Enrique Zozaya-Valdés, Alexandra J. Roth-Schulze, Torsten Thomas

**Affiliations:** Centre for Marine Bio-Innovation and School of Biotechnology and Biomolecular Sciences, The University of New South Wales, SydneyNSW, Australia

**Keywords:** bleaching, microbial shifts, microbial disease, holobiont, environmental stressor, 16S rRNA gene

## Abstract

In recent years, there has been an increase in the rate and severity of diseases affecting habitat-forming marine organisms, such as corals, sponges, and macroalgae. *Delisea pulchra* is a temperate red macroalga that suffers from a bleaching disease that is more frequent during summer, when seawater temperatures are elevated and the alga’s chemical defense is weakened. A bacterial cause for the disease is implied by previous studies showing that some isolated strains can cause bleaching *in vitro* and that host-associated microbial communities are distinct between diseased and healthy individuals. However, nothing is known about the successional events in the microbial community that occur during the development of the disease. To study this aspect in the future, we aimed here to develop an experimental setup to study the bleaching disease in a controllable aquarium environment. Application of a temperature stress (up to 27°C) did not cause a clear and consistent pattern of bleaching, suggesting that temperature alone might not be the only or main factor to cause the disease. The results also showed that the aquarium conditions alone are sufficient to produce bleaching symptoms. Microbial community analysis based on 16S rRNA gene fingerprinting and sequencing showed significant changes after 15 days in the aquarium, indicating that the native microbial associates of *D. pulchra* are not stably maintained. Microbial taxa that were enriched in the aquarium-held *D. pulchra* thalli, however, did not match on a taxonomic level those that have been found to be enriched in natural bleaching events. Together our observations indicate that environmental factors, other than the ones investigated here, might drive the bleaching disease in *D. pulchra* and that the aquarium conditions have substantial impact on the alga-associated microbiome.

## Introduction

Anthropogenic environmental stressors (e.g., pollution and climate change) are increasing the rate and severity of marine diseases worldwide (Harvell et al., 1999, 2002; Webster, 2007; Burge et al., 2014). Particularly concerning is the recent rise in reports of disease outbreaks causing mass mortality to habitat-forming species, such as corals, sponges, and macroalgae. Many diseases have been linked to changes in the microbial communities that live in association with these habitat-formers and in some cases, specific microbial pathogens have been identified (Rosenberg et al., 2007; Webster, 2007; Harvell et al., 2009; Egan et al., 2013). However, for most microbial diseases the interaction between pathogen, host, and environmental factors are not well understood (Egan et al., 2013). There is, however, increasing evidence that environmental stressors, such as elevated seawater temperature, can either increase the virulence or abundance of opportunistic pathogens or weaken the host’s innate defense systems (Harvell et al., 2002, 2009; Webster, 2007; Egan et al., 2013; Burge et al., 2014). It has also been recognized that the health of marine organisms depends on their associated microbial community, which supply nutrients or provide defense against secondary colonization and biofouling. As such a host and associated microorganisms form an entity, termed holobiont, whose function and stability will be influenced by environmental conditions (Rosenberg et al., 2007; Egan et al., 2012; Webster and Taylor, 2012; Fan et al., 2013).

Several macroalgae (seaweeds) suffer from microbial diseases, but there is a general lack of understanding for the mechanistic processes and ecological factors that determine disease (Gachon et al., 2010; Egan et al., 2013). The bleaching disease of the red alga *Delisea pulchra* is arguably one of the best-studied models for macroalgal disease (Egan et al., 2012; Harder et al., 2012). *D. pulchra* possess a chemical defense system against bacterial colonization (Manefield et al., 1999) and invertebrate grazing (Williamson et al., 2004) based on the production of furanone compounds. Furanone concentrations decrease during summer (Wright et al., 2000), when ultra-violet light radiation and temperature are elevated, and this coincides with an increase in the frequency of a disease that is characterized by spot-bleaching of the thallus (Campbell et al., 2011). Experimental reduction of furanone concentration has also been shown to increase the incidence of the bleaching disease (Campbell et al., 2011). The involvement of bacteria in the disease was indicated by two bacterial isolates, *Nautella italica* R11 and *Phaeobacter gallaeciensis* LSS9, being able to cause bleaching in the laboratory, preferentially under conditions where furanone concentrations are decreased and water temperature is increased (Case et al., 2011; Fernandes et al., 2011). Furthermore bleached algae in the field had significantly different microbial communities from healthy individuals (Fernandes et al., 2012; Zozaya-Valdes et al., 2015) and an operational taxonomic units (OTUs) with high similarity to *P. gallaeciensis* LSS9 was significantly enriched in a bleaching event (Zozaya-Valdes et al., 2015). These observations together indicate a link between environmental stressors, weakened host defenses, and bacterial-induced disease, however, how these factors interplay with each others, in particular as a series of events, is not understood.

Time-series experiment under controlled conditions can provide valuable insight into processes of disease development and specific environmental and biological factors that drive it. For example, for the sponge *Rhopaloides odorabile* controlled aquarium experiments have shown that an increase in temperature resulted in reduced expression of symbiotic functions in both the host and its microbial community, which was then followed by the proliferation of opportunistic, scavenging bacteria that ultimately led to tissue necrosis (Fan et al., 2013). In order to study in a similar way the disease in *D. pulchra*, we aimed here to establish a controllable aquarium model for the algae. We used flow-through aquaria to assess the reproducibility and predictability of bleaching symptoms in response to temperature and assessed changes in the microbial communities in response to aquarium conditions with Terminal Restriction Fragment Length Polymorphism (TRFLP) and sequencing of the 16S rRNA gene.

## Materials and Methods

### Temperature-Stress Trials

#### Experimental Design and Set-Up

Two criteria were set to be met for an aquarium model to be suitable to study the effect of environmental stressors on the occurrence of bleaching and its associated microbial community shifts: (1) the algae should remain healthy under control conditions for the duration of the experiment and (2) an environmentally relevant stressor should cause bleaching symptoms in a reproducible and predictable manner. To address these two points, three separate temperature-stress experiments were conducted in an aquarium-laboratory at the Sydney Institute of Marine Science (SIMS).

For these experiments, *D. pulchra* samples were collected by SCUBA at depths of around 9 m at Long Bay, Sydney, Australia, during the austral spring and summer (sampling dates: trail 1: 18/09/2013, trial 2: 31/10/2013, and trial 3: 31/01/2014). All collected individuals were healthy juveniles (∼8.5 cm long thalli) that presented neither visible signs of bleaching nor any atypical phenotypes (e.g., thallus damage). Immediately after collection, thalli were placed in 20 L buckets with fresh seawater for transport to the aquarium-laboratory facilities of SIMS. The aquarium constantly uses fresh seawater that is filtered through a set of hydrocyclones, a set of 100 μm disk filters and a series of bag filters of 50, 25, and 10 μm pore size. The aquarium system is fed by hot- and cold-water sumps (200 L), which take fresh seawater at a rate of approximately 180 L/h and from which the water is mixed for temperature-adjusted fed into individual containers.

For each temperature-stress experiment, individual thalli of *D. pulchra* (*n* = 56, 26, and 28 for trials 1, 2, and 3, respectively) were placed into separate 4 L containers and letting them freely sink to the bottom. Flow-through seawater was delivered at an average constant rate per container of 55 L/h ± 25. Fluorescent light tubes (Sylvania Premium Extra FL36W Cool) were used to supply the algae with an average photosynthetically active radiation (PAR) of 16 μmol photons m^-2^ s^-1^ in a 12 h light:dark cycle. This light intensity was below the average of 103.94 ± 6.96 μmol photons m^-2^ s^-1^ that the algae receive at the collection site at the depth of sampling. All containers were kept at a temperature of 19°C (approximate *in situ* temperature during collection) for one day to let the algae acclimatize. High- (HT) or low-temperature (LT) treatments were randomly assigned to half of the individuals. For the HT treatment during the first two trials, the water was raised to 22°C on the second day and to 25°C on the third day (ramping time of 3 h). This temperature corresponds to the peak temperature found in Sydney waters during summer (Hedge et al., 2014). For the third trial the temperature was raised to 27°C during the third day. The higher temperatures were then kept for the rest of the trial, while the LT treatment remained at 19°C. Trails 1, 2, and 3 were carried out for 6, 5, and 3 weeks, respectively, and measurements and observations were performed on a weekly basis.

#### Physiological Assessment

**FIGURE 1:**
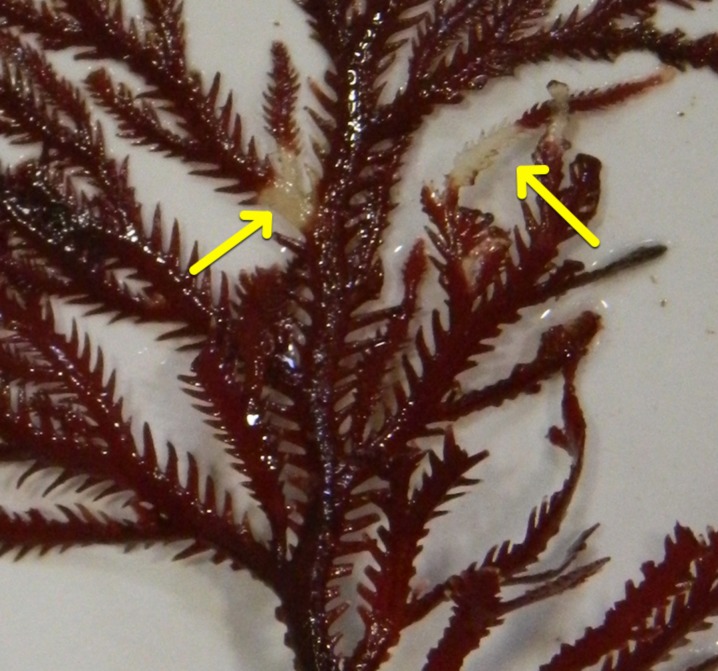
**Example of bleaching phenotype observed in the temperature-stress experiments**.

At time 0 and thereafter every 7 days, each individual was carefully taken out of the container and placed on a light-colored surface to carefully inspect for the appearance of any signs of bleaching. In addition, the algae were photographed and the effective quantum yield of photosynthetic energy conversion (Δ*F*/*F*m′) was measured with a portable pulse amplitude-modulated fluorometer (MINI-PAM photosynthesis yield analyser; Walz, Effeltrich, Germany). As defined in Campbell et al. (2011), an alga was considered bleached, if it had one or more patches of tissue whitening in any part of the thallus (as those showed in **Figure 1**). An alga was considered damaged-by-bleaching, if bleaching observed at a previous time point caused part of the thallus to break off.

### Effect of the Aquarium Environment on Microbial Communities of *Delisea pulchra*

#### Experimental Design

An experiment was carried to assess the possible impact of aquarium-maintenance on the microbial community associated with *D. pulchra*. For this experiment, 12 individual healthy juvenile algae (same size as in the temperature-stress trials) were collected on the 18th of September of 2013 from Long Bay, Sydney, and surface-associated microbial DNA was extracted from half of these (field t0), while the other half was placed in the SIMS aquarium (same set-up and conditions as above). The temperature was kept constant at 17°C, which corresponded to the sea temperature at the time of sampling. After 15 days, six thalli were collected from Long Bay (field t1) and DNA was extracted from these as well as from the thalli that were kept in the aquarium (aquarium t1) following the methods described by Fernandes et al. (2012). Briefly, prior to the extraction, the algal thallus was washed three times with sterilized seawater to remove loosely associated organisms. The DNA extraction was performed by washing the algal thallus (∼1 g) in 20 ml of calcium and magnesium free artificial sea water (CMFASW) supplemented with filter-sterilized and 1:100 diluted Rapid Multi-Enzyme Cleaner (3M, North Ryde, NSW, Australia) for 1h. This was followed by filtering (11 μm pore size) of the supernatant and DNA extraction using phenol: chloroform: isoamyl alcohol (25:24:1) and precipitation of DNA with 0.3 M sodium acetate (pH 5.2) and 0.6 volumes of isopropanol.

#### Terminal Restriction Fragment Length Polymorphism (TRFLP) Analysis

For TRFLP analysis, the 16S rRNA gene was amplified by PCR in a final volume of 40 μl containing 12 μl sterile, de-ionized water, 20 μl EconoTaq Plus Green 2X Master Mix (Lucigen), 2 μl of each of the primers 27F-FAM and 1492R for a final concentration of 2 ng/μl each and 4 μl of DNA template (20 pg/μl). The PCR cycling conditions consisted of: 3 min at 94°C; 30 cycles of 30 s at 94°C, 30 s at 55°C, and 1 min at 72°C, and a final 10 min at 72°C. PCR products were purified using a DNA Clean and Concentrator Kit (Zymo Research) following the manufacturer’s protocol and the DNA was eluted in 30 μl of sterile, de-ionized water. Purified PCR products were digested in 20 μl reactions containing 17 μl purified PCR product, 2 μl 10X restriction buffer and 1 μl *Hae*III. Reactions were incubated at 37°C for 4 h followed by 80°C for 20 min. Digested PCR products were analyzed on an ABI3730 DNA analyzer (Ramaciotti Centre for Genomics, Sydney, NSW, Australia) using the LIZ600 size standard. The raw electropherogram data was processed using the Peak Scanner Software v1.0 (Applied Biosystems) with the peak detection range set to correspond to the size standard used and the minimum peak threshold for the FAM dye, the standard dye and the other dye options set to 1, 500, and 5000, respectively. The resulting table of electropherogram peak information was further processed using T-Rex (Culman et al., 2009) to filter noise with the standard deviation multiplier for the FAM dye set to 3 and to align peaks with a clustering threshold of 0.5. The resulting matrix of samples versus relative peak areas was used in subsequent analysis (see below).

#### 16S rRNA Gene Sequencing and OTU-Based Community Analysis

Three DNA samples from each of the three groups (aquarium t1, field t0, and field t1) were randomly picked for sequencing of the 16S rRNA gene. The V4 region of the 16S rRNA gene was amplified following standard protocols of the Earth Microbiome Project^1^ using the primers 515F and 806R at the Ramaciotti Centre for Genomics. The MiSeq platform was used to generate 151 nucleotide reads from both ends of the amplified products. The sequencing data have been deposited in the Sequence Read Archive (SRA) of the National Center for Biotechnology Information (NCBI) under the study accession SRP065248.

Sequence processing was carried out using the Mothur software v.1.34 (Schloss et al., 2009) and a modified version of the MiSeq standard operational procedure^2^, which can be found in the Supplementary Material. Briefly, paired-end raw reads were merged into contigs of around 250 bp, which were then quality filtered, taxonomically classified and clustered at a sequence identity cutoff of 97% to obtain a ‘sample by OTU’ abundance matrix. In order to reduce the number of spurious OTUs that can pass quality-filtering, OTUs with an overall abundance across samples below 0.0009% (which corresponds to eight sequences per OTU) were removed. Due to the difference in the number of 16S rRNA gene copies that exists between different taxa, the Copyrighter software (Angly et al., 2014) was used to correct the number of reads per OTU as described in Zozaya-Valdes et al. (2015). From the corrected OTU table, the number of OTUs, coverage, Chao1 and the Inverse Simpson diversity index were calculated for 1000 random subsamples using the smallest 16S rRNA gene abundance in any sample (i.e., sample F1-3 with an abundance of 25,297; see **Supplementary Table S2**). The average of the subsamples was used for statistical analysis.

The most abundant sequence for each OTU was used as reference for taxonomic classification. Sequences were classified using the ‘classify.otu’ command in Mothur with default parameters based on three different bacterial 16S rRNA taxonomic outlines, including Silva (release 119), RDP (PDS version 10) and Greengenes (release of August 2013). For the OTUs selected from the statistical analysis, a consensus of the three classifications was manually built by choosing the deepest taxonomic assignment (reporting from the three classifications, only the highest confidence value observed). When different classifications were obtained, alternative taxa are reported.

#### Statistical Analysis

To test for statistically significant differences in the richness (Chao1) and diversity (InvSimpson) between sample groups, Analysis of Variance (ANOVA) was calculated using a one-factor design with three levels: aquarium t1, field t0, and field t1 (overall test in **Tables 1** and **2**). Pairwise tests were also carried out. The data matrices (TRFLP and OTU) were square-root and presence/absence transformed using Primer-E (Clarke and Gorley, 2006) for the analysis of community structure and composition, respectively. Using these data matrices, Bray-Curtis similarities between samples were calculated and clustering patterns between sample types were explored with non-metric multidimensional scaling. Using the same test design as for richness and diversity, hypothesis testing of composition and structure was performed employing PERMANOVA (Clarke and Gorley, 2006) with 9999 permutations.

**Table 1:** TRFLP-based PERMANOVA test results for microbial community structure (i.e., abundances) and composition (i.e., presence/absence) of *D. pulchra*.

	Structure			Composition		
		
Test	pseudo-*F*	*p*-value	unique perms	pseudo-*F*	*p*-value	unique perms
overall test	3.86	0.00005^∗^	85168	3.64	0.0001^∗^	9815
field t0 – field t1	1.11	0.2126	126	1.07	0.2802	126
field t0 – aquarium t1	2.23	0.0048^∗^	210	2.28	0.0054^∗^	210
field t1 – aquarium t1	2.16	0.0022^∗^	462	2.09	0.0021^∗^	462


*The overall test refers to the comparison of the groups field t0, field t1 and aquarium t1. P-values below a significance level of 0.05 are marked with an asterisk.*

**Table 2:** Hypothesis tests of different measurements for the microbial communities of *D. pulchra* based on OTUs obtained from sequencing of the 16S rRNA gene v4 region.

	Chao		InvSimpson		Structure			Composition		
				
Test	*F*	*p*-value	*F*	*p*-value	pseudo-*F*	*p*-value	Unique perms	pseudo-*F*	*p*-value	Unique perms
overall test	12.31	0.0075^∗^	0.04	0.965	4.03	0.0035^∗^	280	3.57	0.0029^∗^	280
field t0 – field t1	5.56	0.0779	–	–	1.46	0.1236	MC	1.42	0.135	MC
field t0 – aquarium t1	42.08	0.0029^∗^	–	–	2.34	0.0172^∗^	MC	2.18	0.0209^∗^	MC
field t1 – aquarium t1	4.57	0.0993	–	–	2.08	0.0281^∗^	MC	1.94	0.0358^∗^	MC


*The overall test refers to the comparison of the groups field t0, field t1, and aquarium t1. For richness (Chao) and diversity (InvSimpson) a conventional ANOVA was used, while for composition (i.e., presence/absence) and structure (i.e., abundances) PERMANOVA tests were performed. *P*-values below a significance level of 0.05 are marked with an asterisk. MC denotes that Monte Carlo simulations where performed in place of permutations.*

Operational taxonomic units that contributed most to the difference between sample types were identified by adjusting the OTU matrix to a multivariate generalized linear model using the Mvabund R package (Wang et al., 2012). Here, each OTU is considered as a variable that was fitted to a separate generalized linear model using a negative binomial distribution. To test for the difference between community structures, the ANOVA function (which implements an analysis of deviance) was applied to the multivariate generalized linear model performing 1000 permutations and with the ‘p.uni’ argument set not to adjust the univariate *p*-values for the family-wise error rate across variables. The univariate ANOVA-like tests were ordered by deviance and the variables (OTUs) that contributed to the top 50% of the overall deviance and that had a *p*-value_unadjusted_ < 0.01 were selected as the highest contributors to the difference between communities. These OTUs were then classified as “unique” (“novel” and “depleted” bars in **Figure 4**), if they were consistently present in all replicates of a group and consistently absent in the other group. The rest of the selected OTUs were classified as “abundant” (“enriched” and “reduced” bars in **Figure 4**) as they could be present in both groups, but were significantly more abundant in one of them.

## Results

### Temperature-Stress Trials

After 1 week of maintenance in the aquarium, we could detect algal thalli that show signs of bleaching (**Figure 1**; for bleaching definition see “Materials and Methods”). Consistent with the visual identification of the bleaching phenotype, the photosynthetic yield of bleached algae was always lower in the bleached tissue (average of 481 sd ±86; **Supplementary Table S1**) compared to healthy tissue (average of 607 sd ±42; **Supplementary Table S1**) and this difference was statistically significant when assessed for trial 1 (*n* = 17, *t* = 6.0614, *df* = 16, *p*-value = 1.65 × 10^-05^ in a paired *t*-test).

**FIGURE 2:**
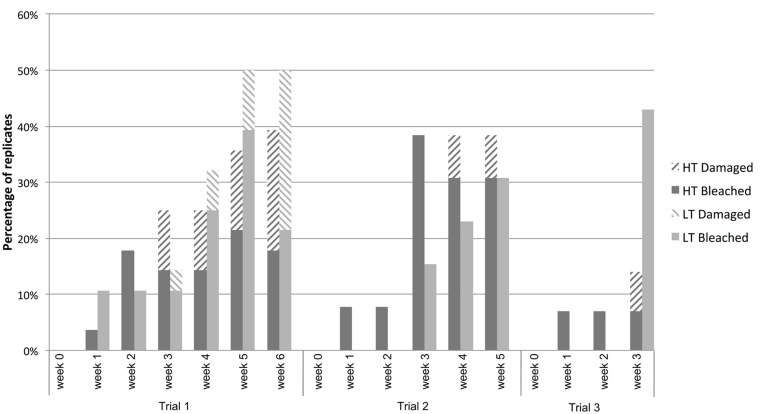
**Percentage of algae that bleached or were damaged-by-bleaching in the high- (HT) and low- (LT) temperature treatments observed in three temperature-stress trials**.

In all three trials the proportion of bleached (including damaged-by-bleaching; for definition see “Materials and Methods”) individuals in the HT treatment increased with time, but never increased above a 40% frequency (**Figure 2**). The number of bleached (including damaged-by-bleaching) individuals in the LT (control) group also increased over time, reaching proportions above the HT group in some of the time points of trials 1 and 3. In addition, the HT treatment had no apparent effect on the photosynthetic yield of healthy tissue of *D. pulchra* compared to the control in any of the trials (see **Supplementary Figure S1**).

This experiment showed that the aquarium conditions alone can produce bleaching in *D. pulchra*, as up to 43% of individuals in a control group developed disease phenotypes even after just 3 weeks (see **Figure 2**). The results also show that high temperatures alone do not result in a reproducible increase in the incidence of bleaching disease above the control level. Therefore the criteria evaluated in the present experiments (see “Materials and Methods”) were not met, which made the current aquarium model not suitable to test the effect of temperature on the incidence of bleaching and the associated changes of the microbial community.

### Effect of Aquarium Conditions on Microbial Community of *D. pulchra*

#### Comparison of Microbial Communities on Field and Aquarium *D. pulchra*

**FIGURE 3:**
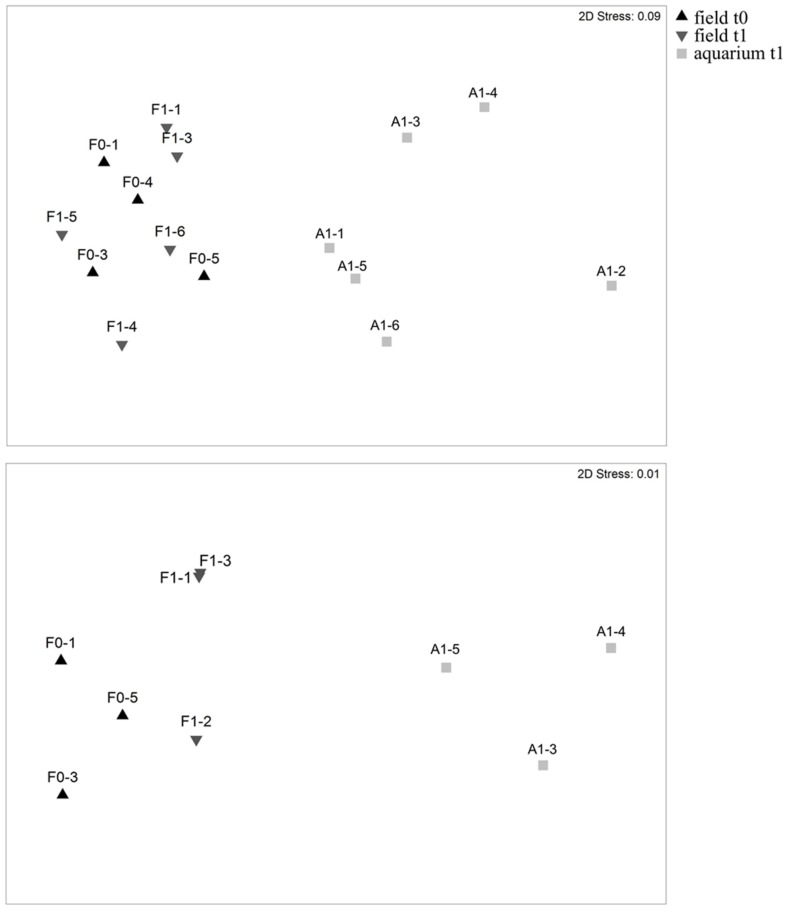
**Non-metric multidimensional scaling based on Bray–Curtis similarity of microbial community structures of *D. pulchra* as assessed by TRFLP fingerprinting **(Upper)** and on v4 region amplicon sequencing **(Lower)** of the 16S rRNA gene.** Samples collected from the field on the first day of sampling are indicated as field t0, while samples collected from the field or the aquarium 15 days after, are indicated as field t1 and aquarium t1, respectively.

The transfer of marine organisms from their natural environment to an aquarium can have an impact on their associated microbial communities (Kooperman et al., 2007; Mohamed et al., 2008a,b; Pratte et al., 2015). To investigate if this also applies to the experimental system used here, we used TRFLP and sequencing of the 16S rRNA gene to analyze the microbial communities on healthy algae that were kept for relatively short times under temperatures that correspond to ambient condition. All thalli collected from the field (field t0 and t1) or after 15 days in the aquarium (aquarium t1) showed no signs of bleaching and were therefore considered healthy. Independent of the analysis method used, Bray–Curtis similarity of community structure (i.e., abundances; **Figure 3**) and composition (i.e., presence/absence; **Supplementary Figure S2**) showed that all field samples (field t0 and field t1) clustered together regardless of the sampling time point and that the field samples clearly separated from the aquarium t1 samples. PERMANOVA hypothesis testing showed that the community composition and structure were significantly different across the three sample types (overall test), but in pairwise comparisons, only between field and aquarium samples (**Tables 1** and **2**). This shows that aquarium maintenance for 15 days can produce significant changes in the microbial communities of *D. pulchra*.

From the sequencing of the 16S rRNA gene we obtained between 25,297 (F1-3) and 66,948 (F0-3) quality-filtered sequences, which clustered into OTUs (at 97% sequence identity) ranging between 566 (A1-3) and 1,068 (F0-3) per sample (see **Supplementary Table S2**). Rarefaction analysis indicated that the sequencing effort covered most of the bacterial diversity present in the samples, which was further supported by Good’s coverage estimates of 99% (**Supplementary Figure S3**, **Supplementary Table S2**). Most field samples showed a greater OTU richness estimate (Chao1) than aquarium samples and ANOVA showed this to be statistically significant in the overall test and between field t0 and aquarium t1 samples (**Supplementary Table S2** and **Table 2**). No statistically significant differences were observed for the inverse Simpson diversity index (**Table 2**).

#### Microbial Community Shift Upon Transfer of *D. pulchra* to the Aquarium: Colonization of Aquarium Bacteria or a Shift of the Natural Community?

**FIGURE 4:**
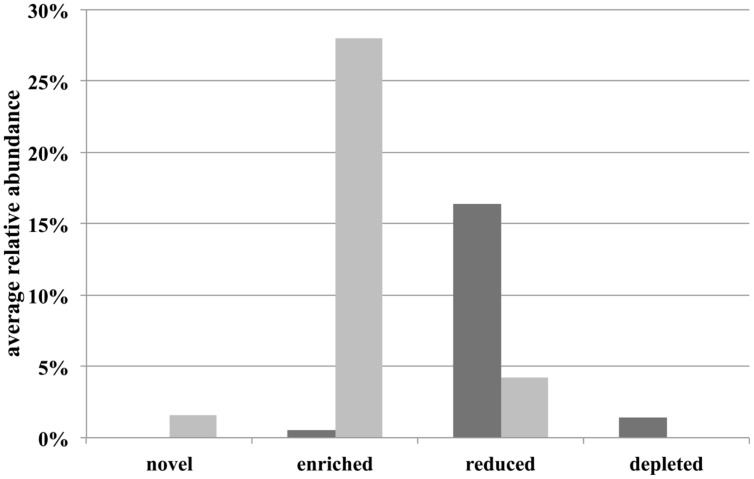
**Average relative abundance in field (dark gray bars) and aquarium samples (light gray bars) of specific OTU classes.** “Novel” and “depleted” are OTUs that are being newly found or not found in aquarium samples compared to field samples.

We then asked if the differences between field- and aquarium-held thalli were due to the colonization and proliferation of non-native bacteria (e.g., contaminants from the aquarium environment) or due to a general shift of the natural microbial community of *D. pulchra*. Fitting of the OTU abundances to a multivariate generalized linear model showed an overall difference between aquarium and field samples (deviance = 5938, *p*-value = 0.01) and identified 95 OTUs that showed a significant individual difference (see “Materials and Methods” and **Supplementary Figure S4**). These OTUs represent 5% of the total OTUs and contribute together to the top 21% of the overall community difference. Of these top contributors, 30 were only found in aquarium samples and could represent potential laboratory contamination (see “novel” group in **Figure 4** and **Supplementary Figure S4**). These OTUs represented only 1.6% of the total community abundance (**Figure 4**). In contrast, the remaining 65 OTUs were present in field samples and thus are considered part of the algae’s natural community. Here, in particular, several OTUs were enriched in aquarium samples to achieve an abundance of 28% or were reduced to levels of 4% (from 16% in filed samples). In summary, these results show that most of the difference between field- and aquarium-held algae was produced by shifts of naturally occurring bacteria and that potential contaminants represent only a small fraction of the community in the aquarium.

Most of differential OTUs were assigned to the phyla Actinobacteria, Bacteroidetes, Planctomycetes, Proteobacteria and Verrucomicrobia (**Supplementary Figure S4**). Analysis of the novel/enriched OTUs at the order level, where most (87%) of the OTUs could still be taxonomically classified, showed that cultivation of *D. pulchra* under aquarium conditions preferentially enriches for certain taxonomic groups of microbial associates (**Supplementary Figure S5**). For example, OTUs assigned to the orders Kordiimonadales, Alteromonadales, and Cytophagales were frequently enriched or novel in microbial communities in the aquarium.

## Discussion

### Effect of Temperature and Aquarium Conditions on the Incidence of *D. pulchra* Bleaching

Infection assays using *D. pulchra* cultured from spores in the laboratory have previously shown that specific bacteria, such as *Nautella italica* R11, can cause bleaching in a temperature-dependent way (Case et al., 2011; Fernandes, 2011). Furthermore seasonal field observations have shown that warmer waters are positively correlated with higher incidence of bleaching in *D. pulchra* (Campbell et al., 2011). However, no experiment has been previously performed to test if temperature alone is sufficient to cause bleaching in *D. pulchra* and therefore we subjected here the algae to a temperature-stress under controlled aquarium conditions.

Increases of temperature to 25°C, which is the peak level observed in the field (Hedge et al., 2014) (trial 1 and 2), or 2°C above it (trial 3), had no clear and reproducible effect on the frequency of bleaching beyond the levels seen in control thalli (i.e., low-temperature treatment). Field observations have shown an overall weak, but statistically significant correlation (*R*^2^ = 0.063, *df* = 565, *P* < 0.005) between bleaching incidence and water temperature for *D. pulchra*, but deeper (7–10 m) populations had no significant correlation (*R*^2^ = 0.006, *df* = 289, *P* < 0.192; Campbell et al., 2011). These field and aquarium results together indicate that temperature alone is not the only or major driver of *D. pulchra* bleaching disease. Solar radiation was also shown to have a very weak correlation with the incidence of bleaching (Campbell et al., 2011). It would thus appear that other factors (e.g., nutrients, physical damage, presence of pathogens, etc.) or combinations thereof need to be investigated to fully understand the conditions that drive disease development and progression.

In the aquarium set-up used here, we also observed that up to half of the *D. pulchra* thalli bleached, even when no temperature stress was applied. Considering the relatively short duration of the experiments (3–6 weeks), this suggests that handling and/or the aquarium conditions impose a stress with consequences for the algae’s health. Transfers to an aquarium have previously been observed to cause stress in other marine sessile organisms, including corals, sponges and macroalgae (Gachon et al., 2010; Luter et al., 2011; Sheridan et al., 2013). In *D. pulchra*, general and environmentally mediated stress has been proposed to negatively impact the furanone-based chemical defense, which could result in bacterial infection (Campbell et al., 2011; Case et al., 2011). Furthermore, variability of furanone concentration have been reported for individual thalli of *D. pulchra* collected from the field (Wright et al., 2000). Together aquarium-mediated stress and between-individual variability could have contributed to the observed bleaching under control conditions and/or the maintenance of health for some individuals in the aquarium after 3–6 weeks, even under high temperatures.

### Microbial Community Changes in Response to the Transfer of *D. pulchra* from a Natural to an Aquarium Setting

One of the factors that could influence the performance of *D. pulchra* is the community of its associated microorganisms and here we observed clear changes in microbial structure, composition, and richness 15 days after transfer to the aquarium. To our knowledge, this is the first time that this has been described for macroalgae, however, similar observations have been made for other marine sessile organisms. For example, using sequencing of 16S rRNA gene clone libraries and denaturing gradient gel electrophoresis (DGGE), Mohamed et al. (2008a,b) observed changes in the abundance of microorganisms associated with sponges *Mycale laxissima* and *Ircinia strobilina* after 6 and 3 months in a flow-through and recirculating aquarium, respectively. Also, Kooperman et al. (2007) found a reduction in the diversity and number of phyla for the microbiota in the surface mucus of the coral *Fungia granulosa* after being held for just 3 weeks in an aquarium with artificial seawater. However, some host-associated communities have also been reported to experience little or no noticeable change when moved from the field to aquaria conditions. For example, DGGE and metagenomic data found that the microbial communities of wild specimens of the sponge *Rhopaloides odorabile* were highly similar to those kept in a flow-through aquarium for up to 4 weeks (Webster et al., 2011; Fan et al., 2013). DGGE analysis also indicated that most of the microbial community of the sponge *Aplysina aerophoba* can remain stable after cultivation under different artificial conditions (Gerçe et al., 2009). The different responses seen in these studies may be explained by the inherent biological properties of the host and its associated microbial communities and their sensitivity or resilience to experimental handling or environmental factors, such as depth, light, nutrients, and salinity. In fact, this kind of environmental factors have all been shown to correlate with shifts in the microbial communities associated to different hosts in natural settings (Guppy and Bythell, 2006; Ainsworth and Hoegh-Guldberg, 2009; Hengst et al., 2010; Meron et al., 2011; Olson and Gao, 2013; Morrow et al., 2015).

As previously proposed for corals (Rosenberg et al., 2007; Bourne et al., 2009), macroalgae and their associated microbial communities should be considered as functional entites or holobionts (Egan et al., 2012). In line with this hypothesis, the observed changes in the microbial communities of *D. pulchra* upon transfer from the field to the aquarium could result in two possible outcomes.

Firstly, changes in the microbial community could cause bacterial infection and this would lead to bleaching, as observed here. The thalli, which were used to test the effect of the aquarium conditions on the microbiota associated with *D. pulchra*, had no bleaching symptoms during the course of the experiment. Further maintenance in the aquarium could have led to bleaching and also additional changes in the microbial community composition. However, a taxonomic comparison with a previous study (Zozaya-Valdes et al., 2015) showed no indication that the microbial communities in the aquarium were shifting toward those seen in bleached *D. pulchra* from the field. Specifically, aquarium conditions favor OTUs belonging to orders such as Kordiimonadales, Alteromonadales, and Cytophagales, while bleached samples on the field were mostly enriched in OTUs assigned to the Flavobacteriales, Rhodobacterales, and Rhizobiales (Zozaya-Valdes et al., 2015; **Supplementary Figure S5**). Bacteria that were enriched under aquarium conditions belong to orders that are commonly found in the marine environment, including those that are in association with sediments and multicellular marine eukaryotes. The Kordiimonadales, for example, are globally distributed alphaproteobacteria, which have been isolated from coastal (Thompson et al., 2011; Yang et al., 2013) and open-ocean waters (Pham et al., 2008; Wang et al., 2010), estuarine and deep-sea sediments (Tian et al., 2008; Li et al., 2009), marine sponges (Anderson et al., 2010), hydrothermal vents (Peng et al., 2010) and from ballast water tanks (Xu et al., 2011). Bacteria belonging to the Kordiimonadales normally occur in their natural habitats at relative abundances below 2% (Xu et al., 2014). Here, however, OTU10 (classified to the genus *Kordiimonas*) had an average relative abundance of 12%, which was the highest for any OTU significantly enriched in algae held under aquarium conditions (**Supplementary Figure S4**). The second most abundant aquarium-enriched OTU (OTU4; 10.4%; **Supplementary Figure S4**) belonged to the genus *Glaciecola* (order Alteromonadales, phylum Gammaproteobacteria), which contains species that have been isolated from diverse marine environments, such as coastal surface seawater (Baik et al., 2006; Chen et al., 2009), arctic ocean seawater (Van Trappen et al., 2004), sea-ice diatom assemblages from Antarctic coasts (Bowman et al., 1998), marine invertebrates (Romanenko et al., 2003), and sea sediments (Matsuyama et al., 2006; Zhang et al., 2006, 2011; Yong et al., 2007). The third and fourth most abundant aquarium-enriched OTUs (OTU32 and OTU56; **Supplementary Figure S4**) are members of the Cytophagales (phylum Bacteroidetes), which are found in coastal environments rich in organic material, such as living or dead macroalgae, aerobic and anaerobic seafloor sediments and decaying marine animals (Reichenbach, 2006).

Secondly, microbial changes could represent an adaptive response of the holobiont to the aquarium environment as has been suggested for corals (Kooperman et al., 2007; Pratte et al., 2015). This outcome is supported by the observation that microbial members of *D. pulchra* are primarily shifting in abundance, rather than being replaced by foreign (e.g., aquarium-derived) microorganisms. If this is true, then the new microbial community, although being different from the ones in the field, could obtain or preserve functions to sustain the health of the host. Future studies therefore need to define the functionality of aquarium-based microbiomes associated with these marine hosts and how they relate to host function in natural situations.

## Author Contributions

EZ-V and TT designed and conceived the research. EZ-V and AJR-S performed the experiments. EZ-V and AJR-S analysed the data. EZ-V and TT wrote the manuscript with input from AJR-S.

## Conflict of Interest Statement

The authors declare that the research was conducted in the absence of any commercial or financial relationships that could be construed as a potential conflict of interest.

## Footnotes

1 http://www.earthmicrobiome.org/emp-standard-protocols/16s/

2 http://www.mothur.org/wiki/MiSeq_SOP
