# Supplementary material for: Effects of Temperature Stress and Aquarium Conditions on the Red Macroalga Delisea pulchra and its Associated Microbial Community
Source: Front Microbiol. 2016 Feb 18;7:161. doi: 10.3389/fmicb.2016.00161 (PMC4757742; doi:10.3389/fmicb.2016.00161)
Supplement: Supplementary file 1 [file Data_Sheet_1.DOCX]

**Supplementary material**

**Effects of temperature stress and aquarium conditions on the red macroalga *Delisea pulchra* and its associated microbial community**

Enrique Zozaya-Valdés, Alexandra J. Roth-Schulze and Torsten Thomas*

Centre for Marine Bio-Innovation, School of Biotechnology and Biomolecular Sciences, The University of New South Wales, Sydney, NSW, Australia

***Correspondence:**

Torsten Thomas, Centre for Marine Bio-Innovation, School of Biotechnology and Biomolecular Sciences, The University of New South Wales, Sydney, NSW 2052, Australia

e-mail: t.thomas@unsw.edu.au

**Mothur protocol for processing of raw reads and creation of an OTU x samples table:**

make.contigs(file=SIMS.files, processors=8)

screen.seqs(fasta=current, group=current, maxambig=0, maxlength=275, contigsreport=SIMS.contigs.report, minoverlap=43, maxhomop=8)

unique.seqs()

count.seqs(name=current, group=current)

align.seqs(fasta=SIMS.trim.contigs.good.unique.fasta, reference=silva.nr_v119.SIMS_v4.align)

screen.seqs(fasta=current, count=current, optimize=start-end, criteria=95)

filter.seqs(fasta=current, vertical=T, trump=.)

unique.seqs(fasta=current, count=current)

pre.cluster(fasta=current, count=current, diffs=2)

chimera.uchime(fasta=current, count=current, dereplicate=t)

remove.seqs(fasta=current, accnos=current)

classify.seqs(fasta=current, count=current, reference=trainset10_082014.pds.fasta, taxonomy=trainset10_082014.pds.tax, cutoff=60)

remove.lineage(fasta=current, count=current, taxonomy=current, taxon=Chloroplast-Mitochondria-unknown-Eukaryota)

cluster.split(fasta=current, count=current, taxonomy=current, splitmethod=classify, taxlevel=4, cutoff=0.15)

make.shared(list=current, count=current, label=0.03)

**Table S1.** Photosynthetic yield measurements of healthy and bleached algae from temperature-stress trial 1.

| **sample id** | **healthy tissue** | **bleached tissue** |
| --- | --- | --- |
| 7 | 615 | 459 |
| 8 | 644 | 564 |
| 9 | 590 | 517 |
| 13 | 563 | 474 |
| 15 | 660 | 623 |
| 20 | 656 | 574 |
| 21 | 621 | 563 |
| 31 | 622 | 412 |
| 33 | 540 | 345 |
| 40 | 529 | 454 |
| 42 | 594 | 534 |
| 45 | 614 | 470 |
| 46 | 672 | 273 |
| 48 | 648 | 504 |
| 53 | 572 | 428 |
| 54 | 613 | 505 |
| 56 | 570 | 481 |
| average | 607 | 481 |
| sd | 42 | 86 |

**Table S2.** Sample sizes and diversity measurements for microbial community analysis of *D. pulchra*. All the data was calculated after performing sequence and OTU quality processing and 16S rRNA gene copy number correction. For all calculations, excepting the number of sequences, the data was normalized to the size of the smallest sample (i.e. F1-3) by random subsampling.

| **sample id** | **type** | **num of seqs** | **num of OTUs** | **coverage** | **chao** | **invsimpson** |
| --- | --- | --- | --- | --- | --- | --- |
| F0-1 | field t0 | 65,870 | 907 | 0.99 | 1,178 | 6.92 |
| F0-3 | field t0 | 66,948 | 1,068 | 0.99 | 1,336 | 20.21 |
| F0-5 | field t0 | 45,133 | 847 | 0.99 | 1,137 | 9.36 |
| F1-1 | field t1 | 43,464 | 682 | 0.99 | 968 | 8.51 |
| F1-2 | field t1 | 38,512 | 858 | 0.99 | 1,126 | 25.41 |
| F1-3 | field t1 | 25,297 | 612 | 0.99 | 855 | 4.21 |
| A1-3 | aquarium t1 | 31,049 | 566 | 0.99 | 784 | 10.56 |
| A1-4 | aquarium t1 | 46,305 | 648 | 0.99 | 834 | 10.95 |
| A1-5 | aquarium t1 | 36,976 | 627 | 0.99 | 818 | 20.29 |

**Figure S1.** Average photosynthetic yield of *D. pulchra* in the low- (LT) and high- temperature (HT) treatments of three temperature stress trials. For each thallus, one measurement was taken and in case the thallus was bleached the reading was taken on healthy tissue. Week 0 refers to the photosynthetic yield at the beginning of the experiment. The error bars represent one standard deviation.

**
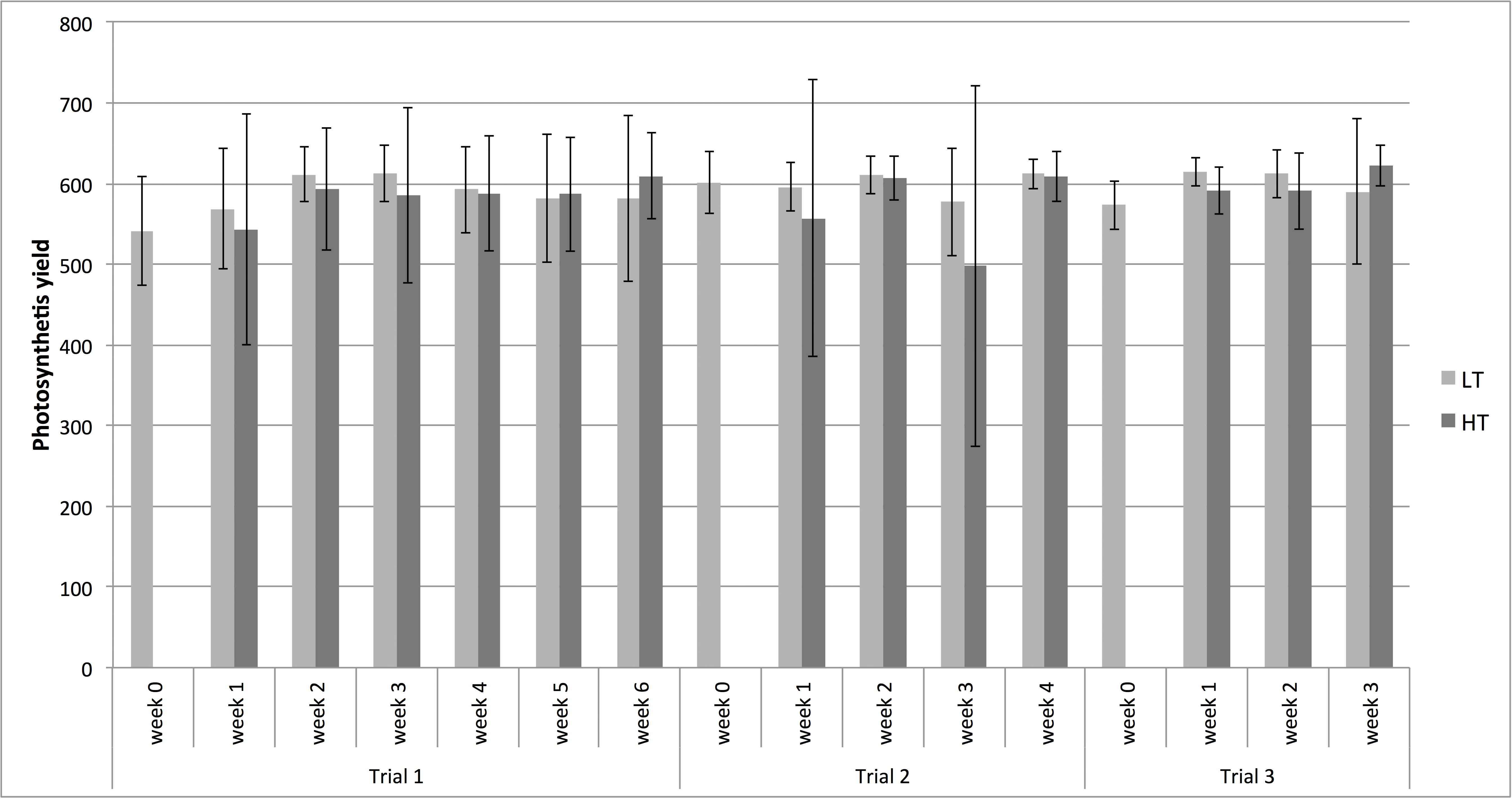
**

**Figure S2.** Non-metric multidimensional scaling based on Bray Curtis similarity of microbial community composition of *D. pulchra* as assessed by TRFLP fingerprinting (upper panel) and amplicon sequencing (lower panel) of the 16S rRNA gene.

**
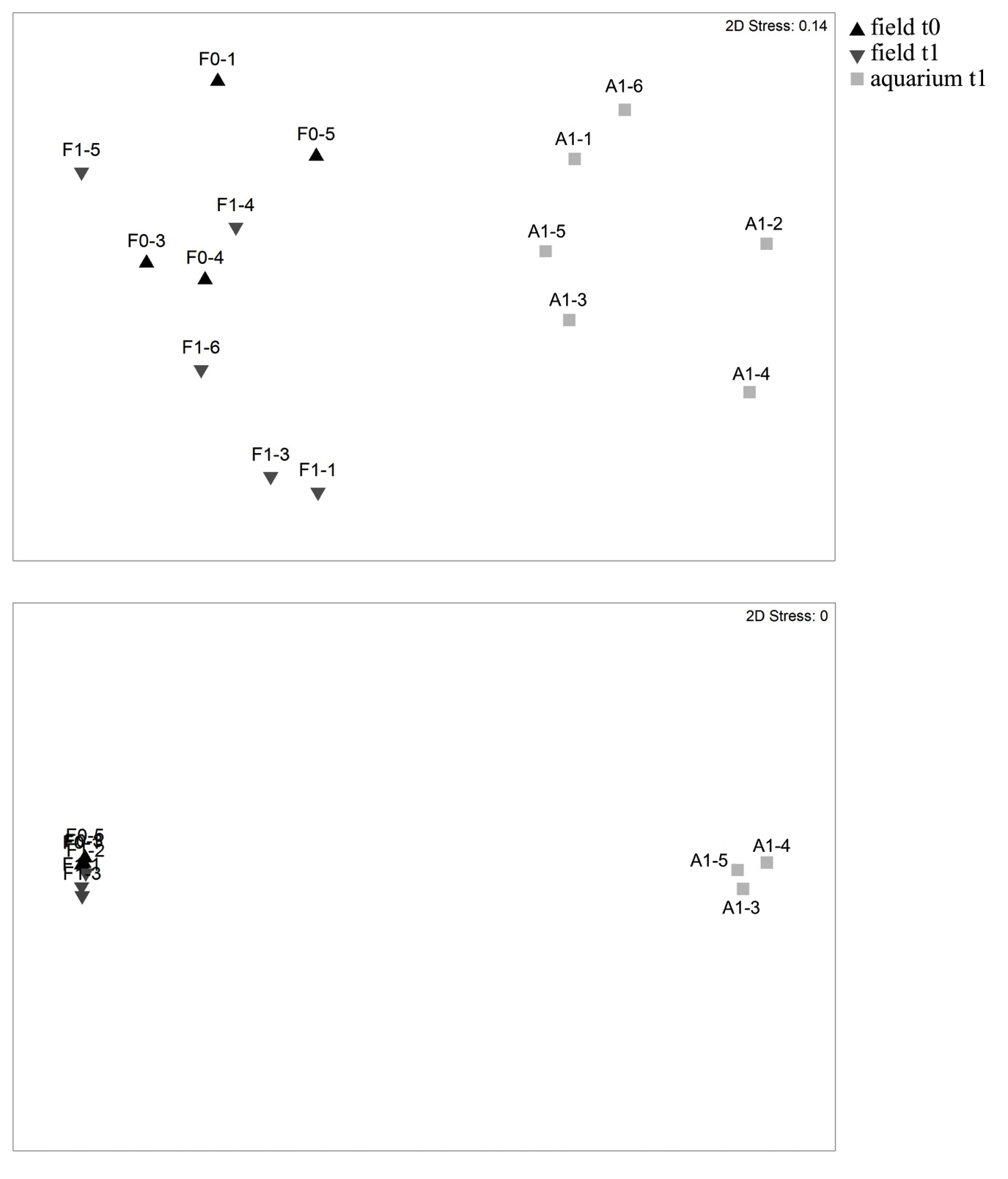
**

**Figure S3.** Rarefaction analysis of *D. pulchra* microbial communities with OTUs defined at a 97% identity and after performing quality filtering, removal of spurious OTUs and 16S rRNA gene copy number correction. The vertical dotted line shows the minimum number of sequences for any sample at which all samples were subsampled for comparative community analysis.

**
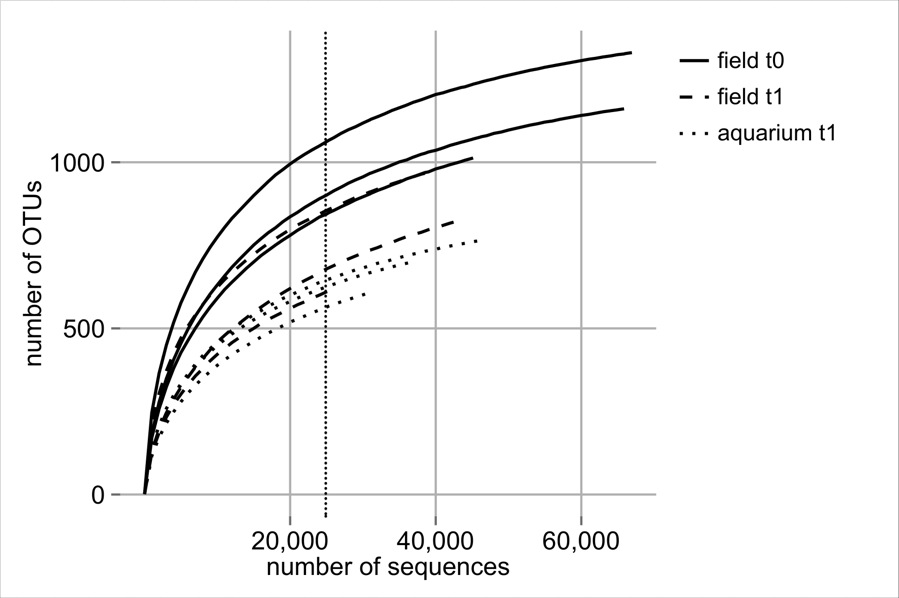
**

**Figure S4.** Relative abundance of OTUs in aquarium (gray boxes) and field (black boxes) samples that were the top contributors to the overall difference between sample types. For OTU selection criteria see Materials and Methods. On the y axis, the capital letter after the colon indicates the taxonomic level, at which the OTU could be classified (D, Domain; P, Phylum; C, Class; O, Order; F, Family; G, Genus). Alternative taxonomic assignment by different databases (RDP, Ribosomal Database Project; GG, Greengenes; S, Silva) are shown in square brackets. The consensus confidence of the OTU classifications are shown in round brackets. The OTUs are ordered in decreasing order from the top by their relative abundance in aquarium samples and were separated in four classes (novel, enriched, reduced and depleted) depending on how they change after 15 days of samples in the aquarium.

**
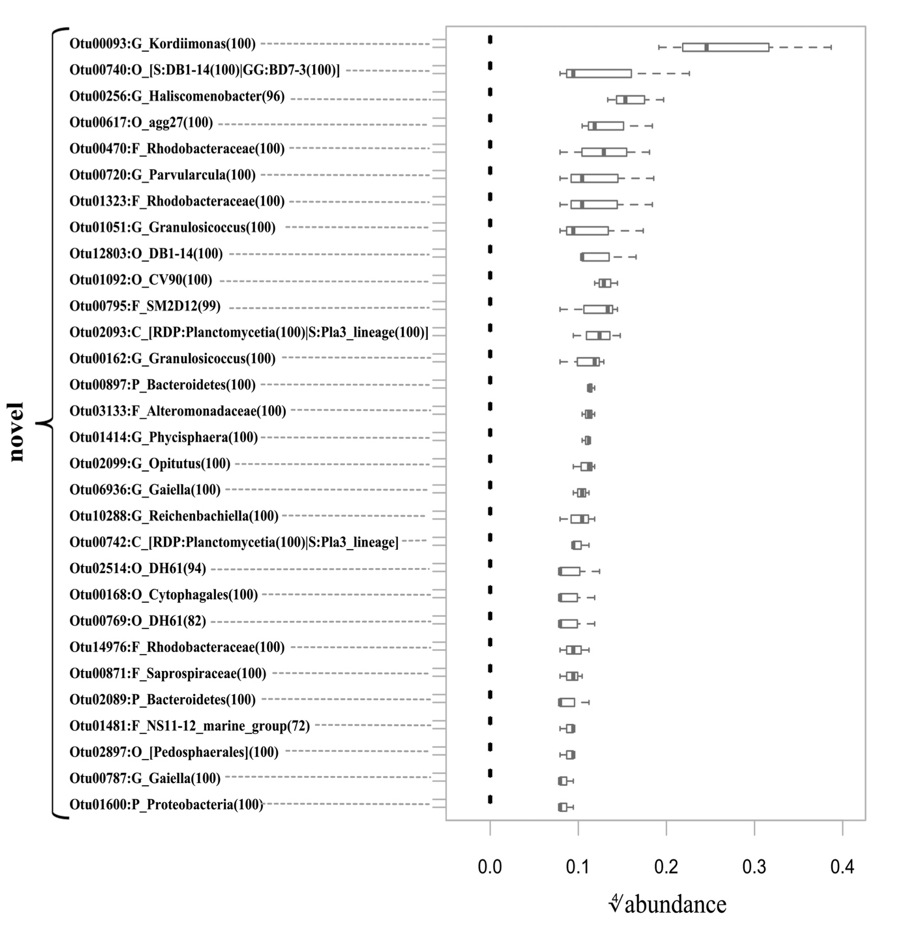
**


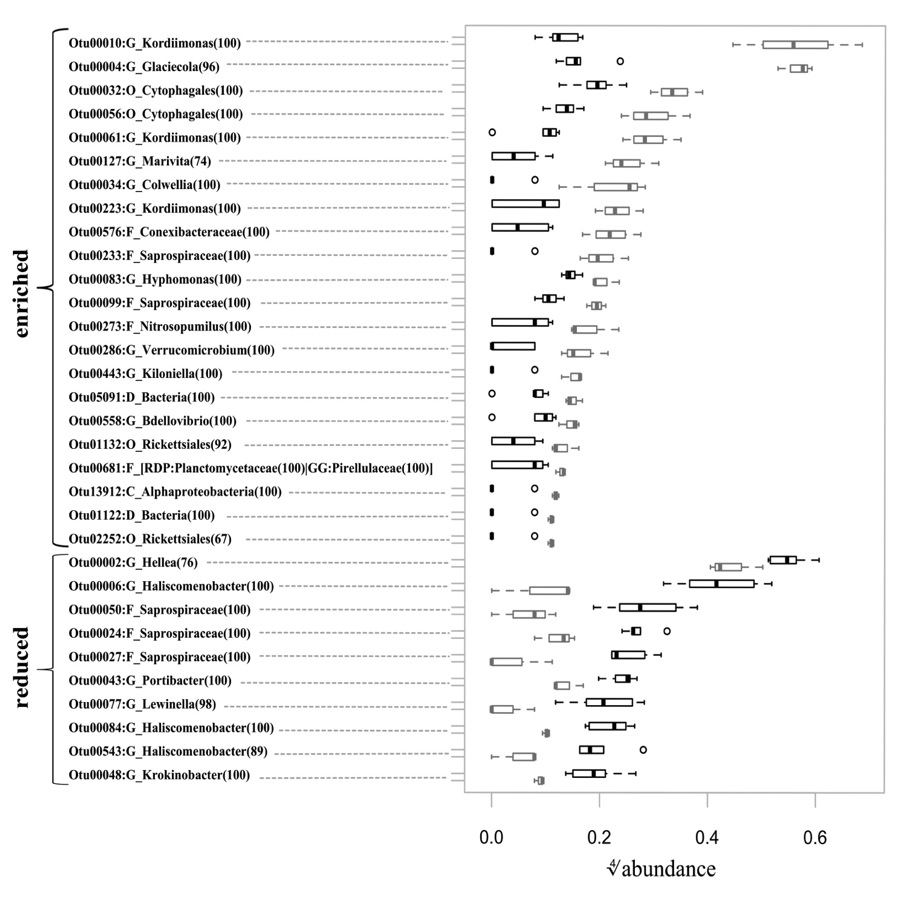


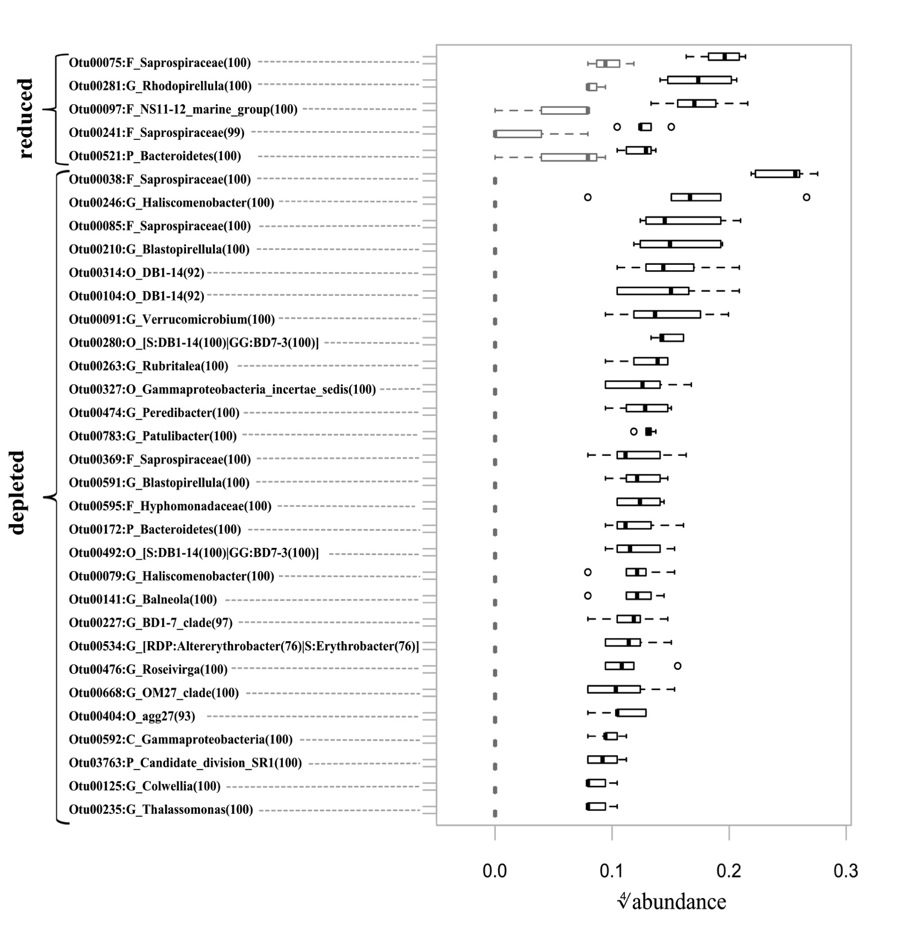


**Figure S5.** Taxonomic distribution of the average relative abundance of novel and significantly enriched OTUs in aquarium samples of present study and in natural bleached samples as found in Zozaya-Valdes et al., 2015. On the y-axis, alternative taxonomic assignment by different databases (RDP, Ribosomal Database Project; GG, Greengenes; S, Silva) are shown in square brackets.

**
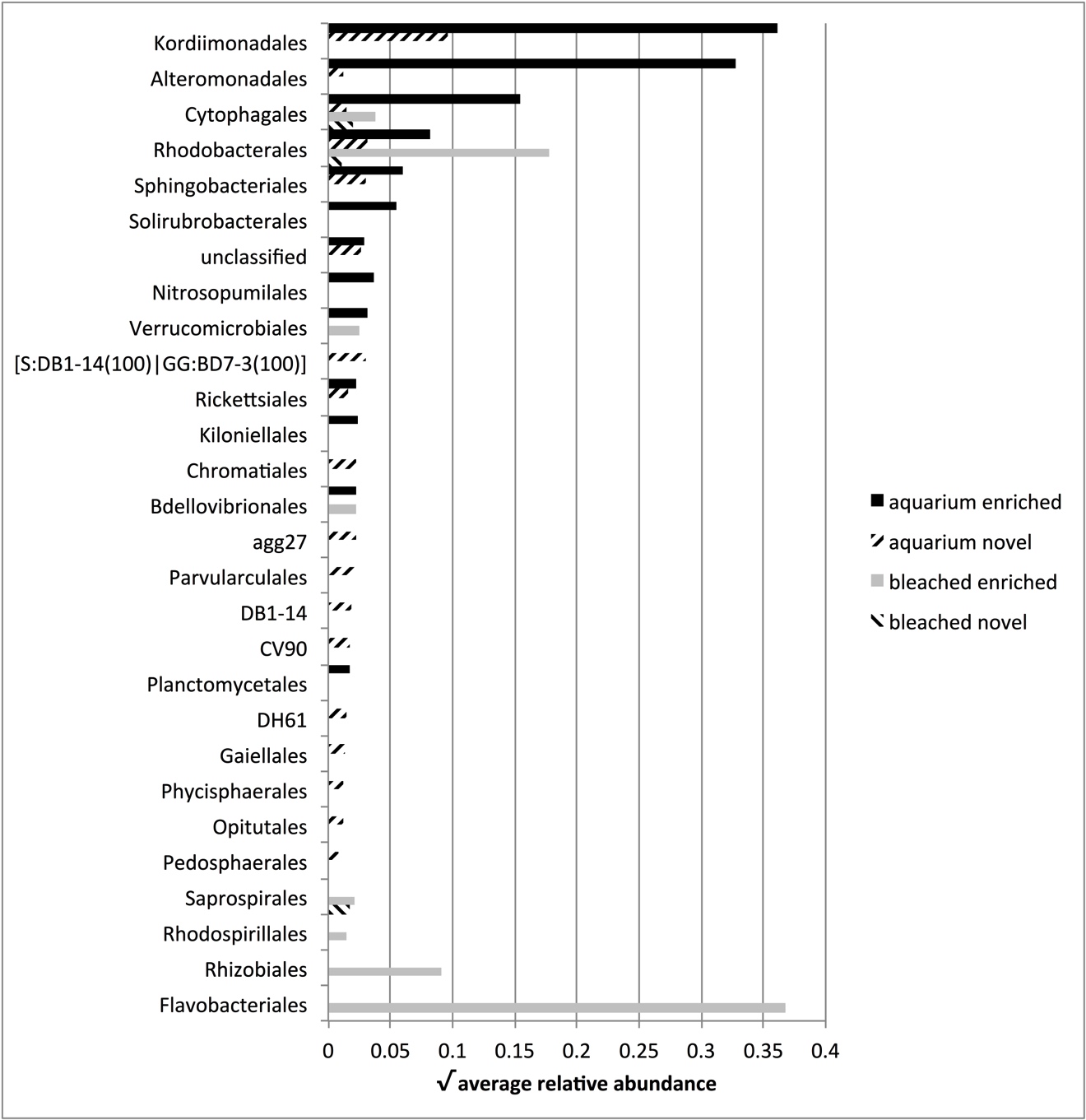
**
